# High seroprevalence of hepatitis E virus in the Free State province of South Africa

**DOI:** 10.4102/sajid.v39i1.577

**Published:** 2024-03-25

**Authors:** Sindiswa S. Maphumulo, Dominique Goedhals, Cornel van Rooyen, Sabeehah Vawda

**Affiliations:** 1 Department of Virology, Faculty of Health Sciences, University of the Free State, Bloemfontein, South Africa; 2 Department of Virology, National Health Laboratory Service, Bloemfontein, South Africa; 3 Department of Virology, PathCare Laboratory, Pretoria, South Africa; 4 Department of Biostatistics, Faculty of Health Sciences, University of the Free State, Bloemfontein, South Africa

**Keywords:** hepatitis E virus, hepatitis E South Africa, hepatitis E seroprevalence, HEV seroepidemiology, viral diseases, viral hepatitis

## Abstract

The seroepidemiology of hepatitis E virus (HEV) in South Africa is limited. We investigated anti-HEV IgM and IgG, in residual hepatitis A, B, and C negative serology specimens, at our public sector Free State (FS) laboratory. Of 299 specimens (01 May–31 October 2020), 182/299 (60.9%) had anti-HEV IgG and 1/299 (0.33%) had anti-HEV IgM. High HEV seroprevalence across different age groups suggests a different epidemiology in the FS, necessitating further research.

**Contribution:**

The need for HEV research in South Africa is highlighted. Clinicians should consider HEV in their differential diagnosis of patients with hepatitis.

## Introduction

Hepatitis E virus (HEV; family: *Hepeviridae*; genus: *Paslahepevirus*) is a global public health concern, causing 20 million hepatitis cases, and 3.3% of viral hepatitis deaths annually.^1,2^ The non-enveloped, positive-stranded RNA virus has four common genotypes (HEV 1 to 4).^2^ Hepatitis E virus 1 (HEV1) and Hepatitis E virus 2 (HEV2) are human-only pathogens, while HEV3 and HEV4 circulate among animals including pigs and rabbits.^2^ Transmission in low-income settings involves primarily HEV1 and to a lesser extent HEV2. These spread mainly via the faecal–oral route via consumption of faeces-contaminated water, causing both sporadic cases and outbreaks. In high-income settings, ingestion of undercooked meat is common, mainly involving HEV3.^2,3^

Hepatitis E virus 1 and HEV2 circulate in many African countries with outbreaks recorded in Namibia and refugee camps in sub-Saharan Africa.^3^ South African HEV seroprevalences ranged from 2% to 42.8% between 1990 and 2020, mainly in two provinces, Gauteng and the Western Cape (WC). Grabow et al. found a 2.1% (16/782) HEV seroprevalence among canoeists and medical students, using an in-house assay,^4^ while Tucker et al. documented 10.7% (*n* = 767) seroprevalence among rural and urban participants in the WC and Eastern Cape (EC), using a commercial assay available at the time.^5^ These older assays were subsequently superseded by better performing assays. Both studies postulated contaminated water consumption as the probable risk factor.^4,5^

In 2013, Andersson et al. described the first case of HEV3 in Southern Africa, in a HIV-positive person from the WC,^6^ while the second case was reported in a WC transplant recipient.^7^ In a more recent seroprevalence study, Madden et al. found a 27.9% (324/1161) seroprevalence in a WC cohort including blood donors. Pork consumption was a significant seropositivity risk factor, and the authors reported a fulminant liver failure case attributed to HEV3.^8^ Another WC study among blood donors conducted by Lopes et al. revealed a 25.3% (78/300) seroprevalence. Hepatitis E virus and hepatitis A virus (HAV) seroprevalences were incongruent, suggesting zoonotic HEV transmission.^9^ Both studies found an increased seroprevalence with age.^8,9^

Korsman et al. found a 29.5% (39/132) HEV seroprevalence among acute hepatitis patients with no identified cause. Anti-HEV IgM was detected in 2/125 specimens, but HEV RNA was undetectable.^10^ Maponga et al. found a 42.8% (107/250) seroprevalence increasing with age among WC blood donors. Additionally, 10 000 donor samples tested for HEV RNA gave a single positive HEV3 sample.^11^ Simani et al. found a low seroprevalence of 3.1% (12/384) among pregnant women in Pretoria, Gauteng.^12^

Worldwide, HEV studies in animals have found that in addition to pigs, farmed cattle, sheep, goats, and rabbits may play a role in the zoonotic spread of HEV to humans. Hepatitis E virus RNA has also been detected in animal milk suggesting the possibility of transmission through raw milk consumption.^13^ In South Africa, animal studies have identified the presence of HEV in pig herds in the EC and WC provinces, with Korsman et al. reporting the presence of HEV RNA in pig-derived food products in Cape Town, suggesting the possibility of food-borne transmission.^14^

The HEV seroprevalence in the Free State is unknown. We investigated HEV seroprevalence in stored patient samples from the Free State province of South Africa, using a commercial enzyme-linked immunosorbent assay (ELISA).

## Methods

Specimens submitted for any viral hepatitis studies to the diagnostic virology laboratory at the National Health Laboratory Service (NHLS), Universitas Academic Hospital (UAH), between 01 May and 31 October 2020, were identified using the laboratory information system (LIS) (*n* = 13 036).

Residual stored specimens of patients in the Free State, irrespective of age, with negative serology for hepatitis A (anti-HAV IgM), B (HBsAg) and C (total anti-HCV) were selected (*n* = 310). Duplicate (*n* = 4), untraceable (*n* = 2), and low volume (*n* = 5) specimens were excluded. A total of 299 specimens with sufficient volume (minimum 100 µL) were included in the study. Demographic details including age, sex, ethnicity, and district were derived from the LIS.

Specimens were tested by ELISA using Fortress Diagnostics HEV-IgM and HEV-IgG ELISA kits (Fortress Diagnostics, United Kingdom) as per manufacturer’s instructions. Positive anti-HEV IgM specimens were retested for HEV IgM as confirmation. Sensitivity and specificity of HEV-IgM as reported in the package insert is 97.1% and 100% respectively. Hepatitis E virus-IgG has a sensitivity of 100% and specificity 86.2%.^15^ Hepatitis E virus immunoassays are generally able to detect antibodies to all four HEV genotypes (HEV1 – HEV4) affecting humans, while discrimination between genotypes requires sequencing.^16^

### Statistical analysis

Analysis was done by the Department of Biostatistics, University of the Free State (UFS), using the Statistical Analysis Software (SAS 9.4). Chi-square was used to assess differences in anti-HEV IgM and anti-HEV IgG positivity rates by age, sex, and district. Continuous variables were summarised by minimum, maximum, or percentiles. Categorical variables were summarised by frequencies and percentages.

### Ethics statement

Ethics approval was obtained from the Health Sciences Research Committee (HSREC) at the University of the Free State. HSREC number: UFS-HSD2020/2071/2411. Patient informed consent was waived as only residual specimens stored in the Division of Virology were utilised. All specimens were de-identified with controlled access to the data-collection spreadsheet to ensure confidentiality.

## Results

Anti-HEV IgG was detected in 182/299 (60.9%) specimens and anti-HEV IgM in one IgG positive specimen (0.3%). Specimens were from five Free State districts (Table 1). Mangaung accounted for the majority (44.5%, 133/299) and a high seropositivity of 62.4% (83/133) (Table 1). Lejweleputswa and Xhariep districts had a high seropositivity of 72.7% (24/33 and 8/11, respectively) but numbers too small to meaningfully analyse (Table 1).

**TABLE 1:** The number of specimens and anti-hepatitis E virus IgG seropositivity per district in the Free State, South Africa, age group and sex.

Variable	Number of specimens	Number of specimens/total specimens, *n* = 299 (%)	Number of positive anti-HEV IgG specimens	
			*n*	%
**District**				
Fezile Dabi	40	13.4	21	52.5
Lejweleputswa	33	11	24	72.7
Mangaung Metro	133	44.5	83	62.4
Thabo Mofutsanyana	81	27.1	46	56.8
Xhariep	11	3.7	8	72.7
Unknown	1	0.3	0	0
**Age group**				
1 (<18 years)	18	6	11	61.1
2 (18–45 years)	156	52.2	92	59
3 (45–64 years)	99	33.1	60	60.6
4 (≥ 65 years)	26	8.7	19	73.1
**Sex**				
Male	109	36.5	69	63.3
Female	190	63.6	113	59.5

HEV, hepatitis E virus.

Patient ages ranged from 0 to 90 years (median 42 years). The specimens were categorised into four age groups: <18, 18–44, 45–64, and ≥65 years. Most specimens were adults in groups 2 (156/299) and 3 (99/299) (Table 1). Group 4 (≥65 years) had the highest seropositivity (73.1%, 19/26). There was no statistically significant difference in seropositivity between the age groups (*p* = 0.65). Most specimens were from females (63.6%, 190/299). More males were anti-HEV IgG seropositive (63.3%, 69/109), but there was no statistical difference in HEV exposure (*p* = 0.81) between the sexes. The ethnicity was unknown in 70.2% (210/299) of specimens.

The positive anti-HEV IgM specimen was of a 62-year-old Caucasian female who presented at the emergency department of a Mangaung hospital. Deranged alanine transaminase (ALT), aspartate transaminase (AST), a conjugated hyperbilirubinaemia, and thrombocytopenia were noted in her laboratory results from the LIS. Anti-HAV IgM, HBsAg, and total anti-HCV were negative with no identifiable cause in other requested laboratory tests available on the LIS. The anti-HEV IgM and anti-HEV IgG optical densities were clearly positive, at 6.6 and 12.5 absorbance to cut-off value (A/C.O) respectively.

## Discussion

Our results indicate a high HEV seroprevalence of 60.9% in the Free State province, higher than elsewhere in South Africa (Table 2) and suggestive of a different epidemiology in the Free State.^4,5,6,7,8,9,10,11,12^ It is unknown if our finding represents the baseline seroprevalence or an increase over the years. The lower seroprevalence in studies from the 1990s may reflect differing serology assay characteristics; however, comparable assay performance in more recent studies cannot account for the differences in seroprevalence.^4,5,15,17^ The difference in seroprevalence could also be influenced by the difference in the target groups included in the studies. Our study focused on patients with suspected hepatitis similar to Korsman et al.,^10^ the only other study of acute hepatitis patients. Seropositivity in our study was more than double that of Korsman’s study.

**TABLE 2:** Hepatitis E virus seroprevalence in studies conducted in South Africa, 2014–2022.

Author	Year published	Region	Population	HEV seroprevalence %
Madden et al.^8^	2014–2015	Western Cape	Medical and Emergency unit inpatients and outpatients + blood donors	27.9 (324/1161)
Lopes et al.^9^	2017	Western Cape	Blood donors	25.3 (78/300)
Korsman et al.^10^	2019	Western Cape	Cases with acute hepatitis	29.5 (39/132)
Maponga et al.^11^	2020	Western Cape	Blood donors	42.8 (107/250)
Simani et al.^12^	2022	Gauteng	Pregnant women	3.1 (12/384)

Note: Please see the full reference list of the article for more information: Maphumulo SS, Goedhals D, Van Rooyen C, Vawda S. High seroprevalence of hepatitis E virus in the Free State province of South Africa. S Afr J Infect Dis. 2024;39(1), a577. https://doi. org/10.4102/sajid.v39i1.577.

HEV, hepatitis E virus.

Most specimens were from the Mangaung district, probably because of our laboratory location within the district and the population size within this district. Therefore, the findings may not be a true reflection of HEV seroepidemiology within the other districts. The details of HEV exposure are unknown, but informal settlements are present in the area and pork is produced throughout the country including the Free State.^18,19^ Pork consumption in South Africa has seen a documented increase of 53% over the last 10 years.^19^ There are no known HEV studies in animals in the Free State; therefore, we cannot comment on the possibility of zoonotic transmission within the province.

The seroprevalence across the age groups was similar, differing from previous studies where increasing seroprevalence with age was documented.^8,9,11^ An increasing seroprevalence with age possibly indicates transmission via the consumption of undercooked pork, suggestive of HEV3. A high seroprevalence across different age groups could indicate transmission via contaminated water, suggestive of HEV1 or HEV2 or transmission via increased consumption of undercooked pork from an early age, indicative of HEV3. Hepatitis E virus 3 transmission via rabbit hunting and consumption may also be a possibility.^13^ A definitive conclusion is not possible because of the small sample size, the unequal number of specimens in the age groups, the lack of information on exposure routes, and the selected study population that includes only those with suspected hepatitis accessing public healthcare.

The mode of transmission and circulating HEV genotype/s within the Free State remain unknown, highlighting the importance of determining the genotype/s of all acute HEV infections within the region. Healthcare worker education on HEV is important to ensure that HEV features in the differential diagnosis of patients presenting with an acute hepatitis, especially when anti-HAV IgM, HBsAg, and anti-HCV are negative. Healthcare worker education on laboratory testing overall is required to reduce inappropriate hepatitis screening.

## Conclusion

The Free State has a high HEV seroprevalence across different age groups, possibly indicating a different epidemiology compared to other provinces in South Africa. The circulating HEV genotype/s in the Free State remain unknown. Healthcare worker education is necessary to ensure appropriate HEV testing. Larger HEV seroprevalence studies including human and animal specimens, inclusive of all provinces are required to determine the seroprevalence of HEV in South Africa. Studies investigating the circulating genotype/s are also needed to further explore the seroepidemiology within the Free State province and contribute to the knowledge of HEV in South Africa.
